# Novel modular chimeric antigen receptor spacer for T cells derived from signal regulatory protein alpha Ig-like domains

**DOI:** 10.3389/fmmed.2022.1049580

**Published:** 2022-12-13

**Authors:** Jan Koski, Farhana Jahan, Annu Luostarinen, Diana Schenkwein, Seppo Ylä-Herttuala, Helka Göös, Hector Monzo, Päivi M. Ojala, Pilvi Maliniemi, Matti Korhonen

**Affiliations:** ^1^ R&D, Finnish Red Cross Blood Service, Helsinki, Finland; ^2^ A.I. Virtanen Institute for Molecular Sciences, University of Eastern Finland, Kuopio, Finland; ^3^ Gene Therapy Unit, Kuopio University Hospital, Kuopio, Finland; ^4^ Translational Cancer Medicine Research Program, University of Helsinki, Helsinki, Finland

**Keywords:** CAR (chimeric antigen receptor), spacer, SIRPA, signal regulatory protein alpha, B-ALL, CD19, FcR

## Abstract

**Background:** T cells equipped with chimeric antigen receptors (CAR) have shown remarkable efficacy in targeting B lineage malignancies. Improvement of the CAR structure is needed, however, with a view to developing flexibly modifiable spacers that are inert in interactions with unwanted cells. Specifically, binding to cells carrying receptors for IgG’s crystallizable fragment (FcR), that recognize IgG-derived domains in CARs is to be avoided.

**Methods:** Two novel CARs targeting the CD19 antigen where the IgG1-CH2 and -CH3 domains were replaced with Ig-like domains from signal-regulatory protein α (SIRPα) were designed *in silico*. An IgG1-based CAR and a CAR lacking both SIRPα and IgG1 domains were used as comparators. The phenotype and memory phenotype of the expanded cells were analyzed by flow cytometry, and CAR T cell activation and cytotoxic efficacy were assessed in co-culture experiments in response to CD19^+^ target cells. Unwanted interactions with FcR-expressing myeloid cells were interrogated in co-culture assays with THP-1 monocytic cells.

**Results:** T cells carrying the novel SIRPα-based CARs enacted potent *in vitro* cytotoxicity against CD19 positive B-lineage leukemia cells, comparable to traditional IgG1-based CAR T cells. Co-culture of IgG1-based CAR T cells with FcR-expressing THP-1 monocytic cells led to prominent cell surface expression of CD69 on T cells together with production of Interleukin (IL)-2 and Interferon-γ, and production of IL-1β, indicating activation of the T cells and monocytes, respectively. Longer co-culture led to killing of the monocytes. No signs of T cell nor monocyte activation were detected in co-cultures of SIRPα-based CAR T cells with THP-1 cells. Arming T cells with the SIRPα-based CARs favored differentiation towards CD4^+^ phenotype during expansion, while the effects on memory phenotype of the T cells were equivalent between the SIRPα- and IgG1-based CARs. In a pilot experiment, T cells modified with one of the SIRPα-based CARs showed dose dependent leukemia cell control.

**Conclusion:** The novel SIRPα based spacers offer a suitable backbone for developing chimeric antigen receptors that evade the off-target binding to FcR while the cells retain a favorable memory phenotype and efficient cytotoxicity, establishing a promising candidate for future *in vivo* and clinical testing.

## 1 Introduction

Chimeric antigen receptor (CAR) based T cell therapies are a novel therapeutic modality for hematological cancers, which have shown remarkable results in the treatment of refractory and relapsed patients with acute lymphoblastic leukemia (ALL) (Maude et al., 2018; Park et al., 2018), diffuse large cell B-cell lymphoma (Turtle et al., 2016; Neelapu et al., 2017; Locke et al., 2019; Lamure et al., 2021) and multiple myeloma (Raje et al., 2019). However, in order to advance the CAR T cell therapy, in addition of selecting the suitable intracellular signaling and co-stimulatory domains, the extracellular CAR structure needs to be fine-tuned to attain highly efficient but tolerable cytotoxicity by preventing redundant interactions initiating possible side-effects. CAR fine-tuning classically includes selection of the intracellular domains e.g., activating CD3ζ domain (1st generation) and preferable co-stimulatory domain CD28, 4-1BB, OX40, CD27 or ICOS (2nd generation) or an activating domain with two co-stimulatory domains (3rd generation). Other ways to fine-tune the CAR are to modify the single chain variable fragment (scFv) by humanizing the sequence, using V_HH_ -binding regions (camelid antibodies; nanobodies), utilizing bi-specific binding, modifying the linker between the binding regions, or replacing the scFv by using artificial protein binding domains or a natural ligand (Larson and Maus, 2021). Along with refining the antigen-binding domain, the structure of the spacer/hinge region between the cell membrane and the antigen-binding scFv requires examination, as it may support or hamper the interaction of the CAR with antigen and effect activation-induced signaling. Commonly used CARs incorporate spacers derived from Immunoglobulin G (IgG) constant heavy chains (CH), from Ig-like molecules [most commonly from CD8α (Alabanza et al., 2017) or CD28 (Kochenderfer et al., 2009b; Alabanza et al., 2017)] or from the extracellular moiety of NGFR (Casucci et al., 2018) or NKG2D (Sentman and Meehan, 2014) respectively.

A critical task in assembling a CAR for functionality *in vivo* is to design a structure that evades the spacer-related off-target interactions while maintaining an optimal activation level and on-target cytotoxicity. The previously used IgG1-based CARs (IgG1-CAR) faced similar hurdles as soluble monoclonal antibodies (mAb) (Gil and Schrum, 2013), namely that the IgG1 heavy chain constant domain-2 (CH2) antibody crystallizable fragment (Fc) binds to Fc-receptor (FcR) expressing myeloid cells, commonly to monocytes/macrophages or NK cells. FcRs are cell surface proteins that facilitate antibody-dependent cell-mediated cytotoxicity and antibody-mediated phagocytosis *via* binding to the Fc-region in antibodies. FcR binding to CARs may lead to myeloid cell activation and inflammation (Almåsbak et al., 2015), CAR T cell activation and destruction of FcR-expressing myeloid cells, sequestration of CAR T cells in the lungs, activation induced cell death (AICD) and overall reduction of CAR T cell activity (Hombach et al., 2010; Almåsbak et al., 2015; Hudecek et al., 2015). Achieving functional CAR T cells *in vivo* requires that the unwanted interactions with off-target cells and the resulting side effects are eliminated.

Here we show that by replacing the IgG1-CH2-CH3 constant domains with signal-regulatory protein α (SIRPα) Ig-like domains, the off-target interactions of CARs can be avoided without compromising the functionality of the CAR T cells.

## 2 Results

In our earlier work we have used a CAR that incorporated as its spacer the CH2 and CH3 domains of IgG1 (Kaartinen et al., 2017; Dufva et al., 2020). However, due to the IgG1 constant domain’s interactions with FcR-expressing cells and the resulting impairment of IgG1-CARs *in vivo* as described above, we reasoned that inert Ig-like domain(s) would be better suited as CAR spacers. To obtain inert and modifiable domains for the CAR platform, we selected elements from SIRPα. SIRPα forms non-covalent dimers *via* its Ig-like C1-type 1 and -2 domains which approximately correspond to the length of IgG1-CH2-CH3 constant domains (IgG1-CH2CH3: ∼9.3 nm; SIRPα-Ig-like C1-domains: ∼8.4 nm; data not shown) and binds the target ligand CD47 only *via* its V-type domain in the N-terminus (Hatherley et al., 2009). We hypothesized that by removing the V-type domain, the remaining SIRPα backbone incorporated within CAR-platform might evade unwanted interactions with other receptors, including the FcR, and provide a modifiable inert universal spacer. Most known CARs are believed to exist as covalently bound dimers linked by disulfide bridges, facilitating co-phosphorylation of intracellular CD3ζ in conjunction with e.g. tyrosine residues of the CD28 domain upon T cell activation induced by the target cells (Stoiber et al., 2019). Facilitating dimer formation in complex CAR structures may be important when designing the spacers from novel components.

### 2.1 Chimeric antigen receptor T cell production and chimeric antigen receptor expression

**FIGURE 1:**
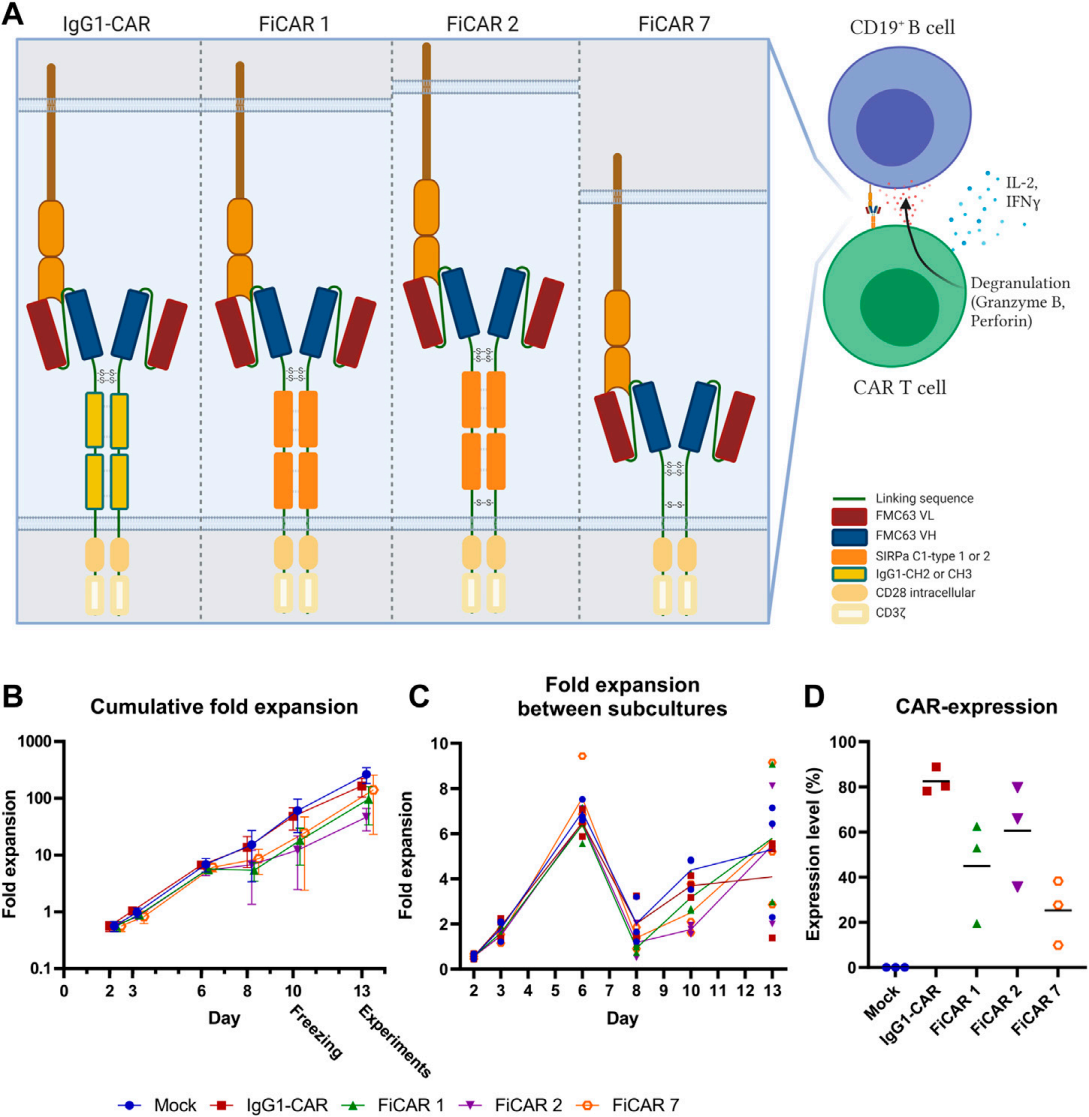
Schematic figure of CARs and T cell expansion kinetics (*n* = 3). **(A)** Estimated CAR structures in a schematic model. The indicated disulfide bridges are hypothetical. **(B)** T cell expansion was assessed with trypan blue and counted with Bio-Rad TC20 Automated Cell Counter on days 2, 3, 6, 8, 10, and 13 prior to subculturing the cells. Results are shown as mean values with standard deviation **(C)** Fold expansion of the cells between subcultures was assessed every 2-3 days. The lines represent mean values of the different CAR T cells. **(D)** CAR expression on day 13 was analyzed by flow cytometry. Results show individual data points and mean values (horizontal lines).

To construct a functional CAR spacer platform, we designed novel spacers that 1) have a modular structure for membrane proximity adjustments and 2) that interact with other cells only *via* the CAR’s scFv domain. To test the functionality of the SIRPα spacer in the CAR platform, we designed two novel CD19 targeting 2^nd^ generation CARs in which the scFv is derived from a CD19 targeting murine monoclonal antibody (FMC63) and the CAR spacer IgG1 constant domains are replaced with Ig-like domains from SIRPα (FiCAR 1). In addition, our second structure includes a juxtamembrane cysteine from CD28 (FiCAR 2) that may enhance covalent dimerization of the CAR. For signaling both CARs contain transmembrane and intracellular CD28 and intracellular CD3ζ domains. As a control we used CD19-IgG1(CH2-CH3)-CD28ζ (here called IgG1-CAR), CD19−CD28ζ (FiCAR 7) CARs (Figure 1A) and an empty (mock) vector. CARs were transduced into T cells using a third generation lentiviral vector where the expression of the CAR is driven by the hPGK-promoter (Schenkwein et al., 2010).

The T cells expanded 48-260 fold within 13 days, with FiCAR 2 transduced cells showing a non-significant tendency for slower growth (Figure 1B). All FiCAR T cells appear to have a characteristic second peak in growth (Figure 1C) after the cells were thawed on day 10.25.3%–88.8% of the T cells expressed CARs as analyzed by flow cytometry on day 13 in different cultures (mean ± SD; IgG1-CAR 88.8 ± 5.6, FiCAR 1 45.0 ± 22.6, FiCAR 2 60.6 ± 22.6 and FiCAR 7 25.3 ± 14.3) as measured by subtracting the CAR antibody binding results of mock T cells (13.25 ± 5.2) (Figure 1D and Supplementary Figure S1B). Representative gating strategies are shown in Supplementary Figure S2.

### 2.2 The different chimeric antigen receptors have equivalent effects on the memory phenotype and maturation of T cells

**FIGURE 2:**
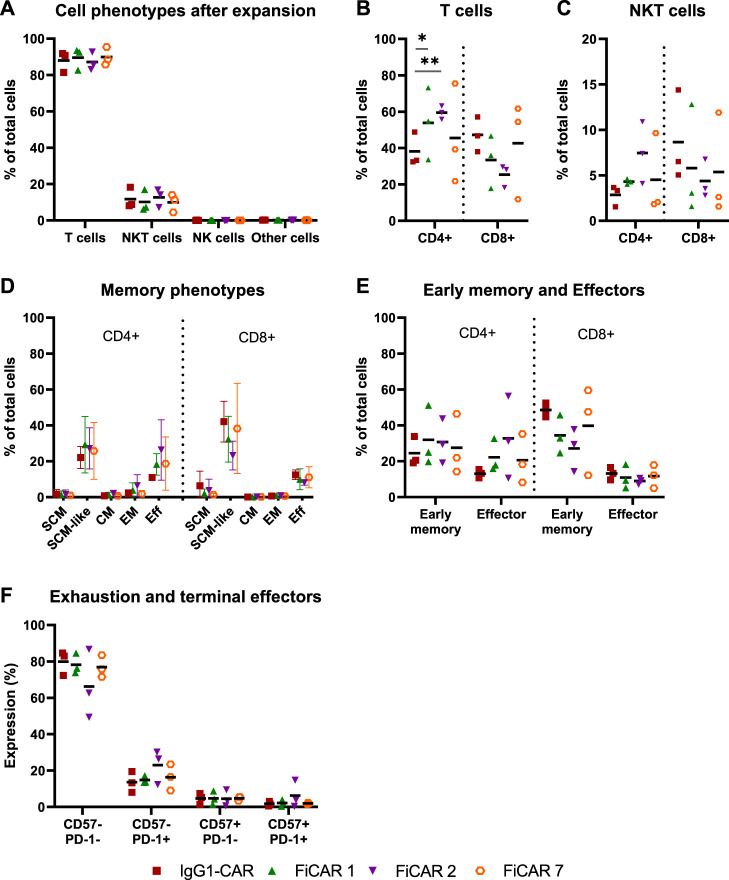
Characterization of cell phenotypes after expansion. T cell products (*n* = 3) were expanded for 13 days and their phenotypes analyzed by flow cytometry. Results are shown as individual data points with mean values **(A–C,E,F)** or mean values with minimum and maximum values **(D)**. **(A)** Cell phenotypes were determined with the following antibody combinations: T cells CD3^+^CD56^−^; NKT cells CD3^+^CD56^+^; NK cells CD3^−^CD56^+^ and other cells CD3^−^CD56^−^. **(B,C)** The proportions of CD4 and CD8 positive cells in T cell and NKT cell populations. Statistical significance was calculated using Mann-Whitney test (*p < 0.05; **p < 0.001). **(D)** On day 13 of the expansion, cells were analyzed for memory phenotypes. **(E)** SCM, SCM-like and CM memory phenotypes were grouped together as an “early memory phenotype” group and EM and Eff as an “effector phenotype” group. **(F)** The cells were analyzed for the exhausted (PD-1 positive) and terminally differentiated (CD57 positive) groups.

The phenotypes and memory phenotypes of the CAR T cell batches were analyzed after 13 days of expansion. The majority of the cells were T cells (CD3^+^ CD56^−^; 86-90%), the rest being NKT cells (CD3^+^ CD56^+^; 9-13%), with very little additional contribution by NK cells or residual CD3^−^ CD56^−^ cells (Figure 2A). By day 13 of expansion, T cells armed with the SIRPα-based FiCARs 1 (*p* = 0.012) and 2 (*p* < 0.001) favored differentiation towards CD4^+^ phenotype compared to IgG1-CAR (Figure 2B). In NKT cells the CD4^+^ phenotype was prominent only in FiCAR 2 -transduced T cells (Figure 2C).

Earlier we reported that the concentration of IL-2 during CAR T cell expansion influences T cell memory phenotype (Kaartinen et al., 2017). Accordingly, we used 100 U/ml of IL-2 in CAR T cell production to prevent excessive differentiation and maturation of the T cells. The T cell memory phenotypes of the expanded cells are shown in Figure 2D. To better distinguish the effects of the expansion process and various CARs on the memory phenotype, we grouped (Table 1.) the various T cell memory subgroups into Early memory (=T_scm_, T_scm-like_ and T_cm_) and Effector (=T_em_ and T_eff_) groups (Figure 2E). The different CARs had no significant effect on the memory phenotype. Furthermore, different CAR T cells had similar overall exhaustion levels as measured using PD-1 surface expression together with the T cell terminal effector maturation-associated marker CD57, although we observed a non-significant increase in the expression of PD-1 and CD57 in the FiCAR2 T cells. After a 13-day expansion, in all different CAR-transduced T cells most of the CD4 and CD8 cells were negative for CD57 and PD-1 (66.2-79.9%), with a minority expressing one or both surface markers (CD57^−^ PD-1^+^: 8-30%; CD57^+^ PD-1^-^: 0.6-9.4%; CD57^+^ PD-1^+^: 0.4-14.7; Figure 2F).

**TABLE 1:** Cell surface marker expression patterns used for cell phenotype and T cell memory phenotype analysis.


Cell phenotypes						
T cells			CD3+	CD56−		
NKT cells			CD3+	CD56+		
NK cells			CD3−	CD56+		
Other cells			CD3−	CD56−		
Memory phenotypes						
Naïve	Naïve	CD95−	CD45RO−	CD45RA+	CD27+
Memory stem cell	SCM	Early memory^a^	CD95+	CD45RO−	CD45RA+	CD27+
Stem cell-like	SCM-like		CD95+	CD45RO+	CD45RA+	CD27+
Central memory	CM		CD95+	CD45RO+	CD45RA−	CD27+
Effector memory	EM	Effector^b^	CD95+	CD45RO+	CD45RA−	CD27−
Effector	Eff		CD95+	CD45RO+	CD45RA+	CD27−

^a^ Combined “Early memory” group.

^b^ Combined “Effector” group.

### 2.3 FiCARs induce equal on-target activation and cytotoxic functionality in T cells compared with conventional IgG1-chimeric antigen receptor

**FIGURE 3:**
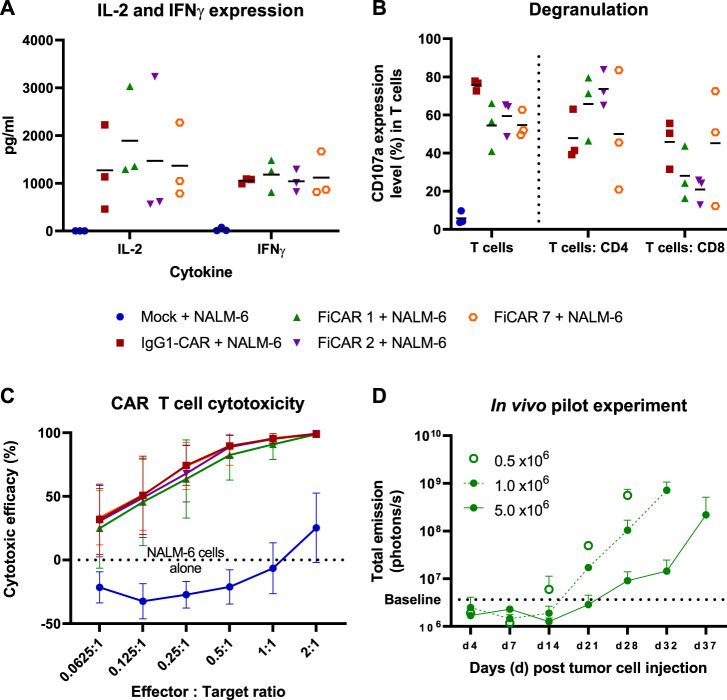
T cell responses and cytotoxicity against CD19 positive NALM-6 cells. The mean (black horizontal lines) and individual data points are shown [Figures **(A,B)**; *n* = 3] **(A)** CAR T cells were co-cultured with NALM-6 cells at 1:1 E:T ratio for 18 h after which the cytokines were analyzed from co-culture supernatants. **(B)** Degranulation of T cells in response to CD19 positive NALM -6 cells was analyzed by staining the CD107a in T cells after 4 h co-culture in the presence on GolgiStop protein transport inhibitor. The results show the percentage of CD107a expressing cells of total T cells and of the CD4 and CD8 subpopulations. **(C)** *In vitro* cytotoxicity of CAR T cells against luciferase expressing CD19^+^ NALM -6 cells at various E:T ratios. The mean±SD is shown. **(D)** *In vivo* pilot experiment. 0.5 x 10^6^ Luc^+^ NALM -6 cells were engrafted *via* tail vein into NSG mice (*n* = 2 per group) on day 0. The indicated number of CAR T cells were injected on day 7. The mice were imaged between days 4-37 and the total emission from luciferase expressing CD19^+^ NALM -6 cells was measured. The values present mean +SD.

Having established that T cells carrying the SIRPα-based CARs can successfully be generated, we next analyzed the functional characteristics of the CAR T cells in response to target-dependent activation. To analyze CAR T cell activation against CD19^+^ NALM-6 cells, we measured cytokine production in overnight co-cultures at a 1:1 effector:target (E:T) cell -ratio (Figure 3A). All the T cells expressing different CARs produced similar amounts of IL-2, with a non-significant trend of higher IL-2 production (∼1.3 fold more) by FiCAR 1 -carrying cells. No differences in IFNγ production were observed.

We then investigated the ability of the T cells to release cytotoxic granules in response to a 4 h co-culture with CD19^+^ target cells by measuring the cell surface expression of lysosomal-associated membrane protein-1 (CD107a). The fraction of degranulating T cells in response to target cells paralleled the proportion of CAR-expressing cells in each culture (Figure 3B), confirming the functionality of CAR -expressing cells. In FiCAR 1 and -2 T cell cultures, a relatively higher proportion of CD4^+^CD107a^+^ cells were detected, and conversely the IgG1-CAR and FiCAR 7 CAR T cell cultures carried more CD8^+^ T cells that were degranulating. This difference corresponded to the higher CD4^+^ T cell content in the FiCAR 1 and -2 T cell cultures (Figure 3B).

Despite the differing CAR expression levels and CD8^+^ cell numbers in the degranulation assay, in an 18 h co-culture experiment with CD19^+^ target cells, all CAR T cells displayed remarkably similar cytotoxicity against NALM-6 cell targets (Figure 3C). All CAR T cells demonstrated 100% killing efficacy at a 2:1 (E:T) ratio and similar efficiencies also at lower E:T ratios. Cytotoxicity experiments against two other CD19^+^ ALL cell lines (Kasumi-2 and RS4.11.) showed similar results (Supplementary Figures S1C-D).

To further assess the functionality of the FiCAR T cells, we set up a small-scale pilot study to investigate the efficacy of FiCAR 1 T cells against established NALM-6 cell leukemia in a NOD. Cg-Prkdc^scid^ Il2rg^tm1Wjl^/SzJ (NSG) mouse model using different doses of FiCAR 1 T cells. FiCAR 1 T cells showed dose dependent leukemia cell control indicating efficient functionality (Figure 3D). However, as the pilot study only included a small number of mice and no negative control T cells, more extensive studies are required to conclude if FiCAR 1 T cells are functional *in vivo* (Koski J. et al., manuscript in preparation).

### 2.4 FiCAR T cells evade off-target activation by myeloid cells

**FIGURE 4:**
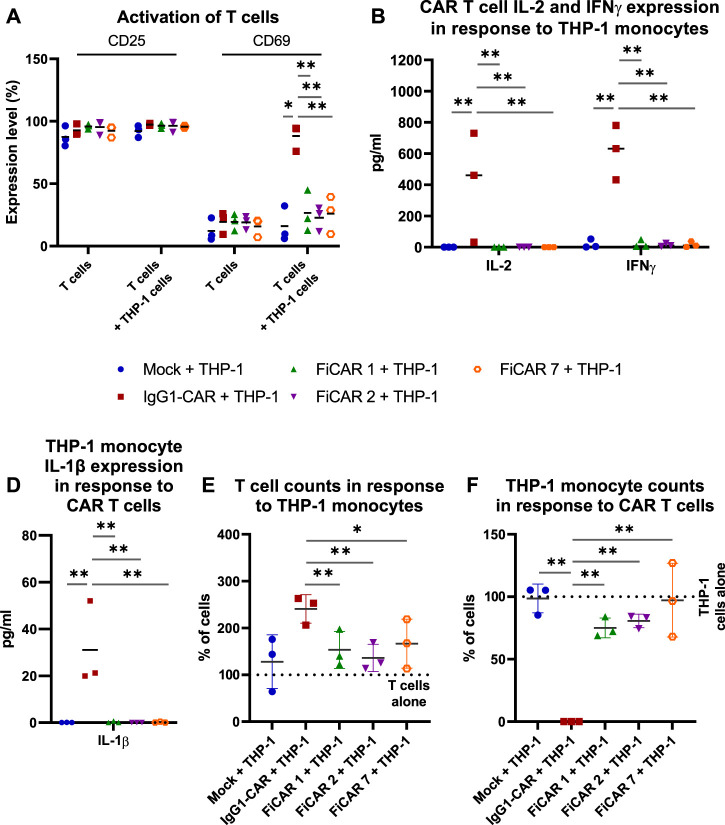
CAR T cell interactions with FcR-expressing THP-1 monocytes. CAR T cells were co-cultured with monocytes at a 1:1 (effector cell: off-target cell) ratio (*n* = 3) for 18 h **(A–C)** or 48 h **(D,E)**. The activation of CAR T cells was measured by analyzing expression of cell surface activation markers [**(A)** CD25, CD69; flow cytometry] and by measuring the CAR T cell and monocyte activation induced cytokines using a flow cytometry-based CBA array [**(B)** CAR T cells: IFNγ and IL-2; **(C)** monocytes: IL-1β]. **(D–E)**: T cells **(D)** and THP-1 monocytes **(E)** were quantified by flow cytometry after co-culture for 48 h. 100% denotes the number of T cells **(D)** or THP-1 cells **(E)** cultured alone. The Mann-Whitney test **(A,D,E)** and one-way ANOVA with Bonferroni correction for multiple comparisons **(B,C)** were assessed for calculating the statistical significances (*p < 0.05; **p < 0.001).

After confirming the cytotoxic functionality of SIRPα-based CARs, we evaluated the interactions of CAR T cell with myeloid cells by co-culturing CAR T cells with a FcR-expressing THP-1 monocytic cell line (Fleit and Kobasiuk, 1991) at a 1:1 (effector: off-target cell; E:OT) ratio for 18 h. Cell activation by the co-culture was measured by staining for the interleukin-2 receptor alpha chain (CD25) and C-Type lectin protein (CD69) that indicate long-term and short-term activation (González-Amaro et al., 2013; Shatrova et al., 2016) of T cells, respectively (Figure 4A), and by measuring cytokines produced by T cells (Figure 4B; CAR T cells: IFNγ and IL-2) and monocytes in response to CAR-dependent activation (Figure 4C; monocytes: IL-1β). All the CAR T cells expressed high and equivalent levels of the CD25 activation marker with or without THP-1 monocytes, which likely resulted from activation by the CD3/CD28 microbeads and supplemental IL-2 during CAR T cell production. However, in co-cultures with CAR T cells and THP-1 monocytes, only the IgG1-CAR T cells expressed high levels of cell surface CD69 in contrast to FiCAR-transduced T cells (*p* < 0.001 for all FiCARs) and Mock T cells (*p* = 0.024). Correspondingly, only the IgG1-CAR T cells produced activation-induced cytokines IL-2 and IFNγ (FiCAR 1 vs. IgG1-CAR, IL-2: *p* < 0.001; IFNγ: *p* < 0.001 and FiCAR 2 vs. IgG1-CAR, IL-2: *p* < 0.001; IFNγ: *p* < 0.001) in co-cultures with THP-1 cells, and only the IgG1-CAR T cells induced production of IL-1β (FiCAR 1 and FiCAR 2 vs. IgG1-CAR, *p* < 0.001) in monocytes. In addition to activation, after a 48 h co-culture of CAR T cells with THP-1 monocytes, we observed a higher proliferation by IgG1-CAR T cells (Figure 4D; Mock, *p* = 0.064; FiCAR 1, *p* < 0.001; FICAR 2, *p* < 0.001 and FiCAR 7 T cells, *p* = 0.001), and complete absence of THP-1 monocytes in contrast to co-cultures of THP-1 monocytes with Mock (*p* < 0.001) or FiCAR 1 (*p* < 0.001), FiCAR 2 (*p* < 0.001) or FiCAR 7 (*p* < 0.001) T cells (Figure 4E). Taken together, these data show that co-culture of FcR-carrying monocytes with IgG1-CAR T cells leads to T cells and monocyte activation, and killing of the monocytes by the T cells, with a phenomenon not observed in co-cultures of FiCAR T cells with monocytes.

## 3 Discussion

CAR T cell therapy is being developed along a wide front and has been approved for therapy of B-lineage ALL and lymphomas as well as multiple myeloma. Nevertheless, more thorough structural optimization of the CAR itself is still needed to improve the function of the CAR T cells, in addition to developing methods to achieve optimal T cell memory phenotype for persistent and potent CAR T cells. Fine-tuning the spacer/hinge region of CARs remains sparsely studied, and its detailed examination may yield important insights into optimizing the CAR structure (Leick et al., 2022).

The classical IgG1-based spacer used in CARs consists of two Ig-domains, CH2 and CH3, derived from the constant region of the heavy chain (Hombach et al., 2010; Almåsbak et al., 2015; Hudecek et al., 2015; Watanabe et al., 2016). We reasoned that, to substitute for the IgG1 CH-domains, a similar spacer structure might be advantageous. SIRPα is a myeloid cell membrane receptor that interacts with the membrane protein CD47, which acts as a marker of self and negatively regulates phagocytosis *via* SIRPα (Hatherley et al., 2009). As the IgG1-CH2CH3 region (Yang et al., 2018), SIRPα is a non-covalently dimerizing transmembrane receptor consisting three extracellular Ig-like domains: the Ig-like C1-type 2, Ig-like C1-type 1, and Ig-like V-type domains (direction from C′ to N′) (Hatherley et al., 2009), of which the latter binds CD47. SIRPα has no other known interactions (Hatherley et al., 2007). We hypothesized that the Ig-like C1-type 1 and type 2 domains could be used in FiCARs 1 and -2 as substitutes for the IgG1-CH2 and CH3 domains in the CAR spacer, comprising similar spatial dimensions without structural interactions with other molecules.

Second, the hinge region of IgG1 contains two cysteine residues that form the two disulfide bridges between the heavy chains in IgG1 (Harris et al., 1998), and it is believed that this covalent dimerization also occurs in CARs containing the IgG1 derived hinge (Van Der Stegen et al., 2015). We thus incorporated the hinge of the CD19 targeting monoclonal antibody FMC63 into FiCAR 1, -2 and -7. However, the exact functionality of disulfide bridging for CAR function remains uncharted. Thus, in designing FiCAR 2, we asked the question: would the FiCAR structure benefit from additional stabilization near the cell membrane, and could this be accomplished by a third disulfide bridge? To effect this, we utilized the juxtamembrane spacer region of CD28, that contains a cysteine residue that effects dimerization of CD28 (Evans et al., 2005) in constructing FiCAR 2. To create the ‘simple’ CAR structure FiCAR 7, we modified a published CAR sequence (Kochenderfer et al., 2009a) by including the same FMC63 hinge and the same juxtamembrane spacer region from CD28 that was employed in FiCAR 2.

All CARs were successfully expressed on T cells, however, after day 6 of culture the expansion rate of the FiCAR T cells appeared to be somewhat (non-significantly) lower than that of IgG1-CAR and mock T cells (Figure 1C). After the expansion, ∼90% of the cells were T cells and ∼10% were NKT cells as we have reported previously (Kaartinen et al., 2017). The CD4^+^:CD8^+^ -ratios were donor-dependent, but FiCAR 1 and -2 T cell expansions produced somewhat more CD4^+^ cells compared to IgG1-CAR (Figure 2B) -transduced T cells, demonstrating the impact of the CAR spacer in T cells. The difference in CD4:CD8 -ratios suggests antigen-independent signaling favoring the CD4^+^ T cells formation or AICD in the CD8^+^ T cells. However, no significant target-independent signaling was seen in our studies using Jurkat T cells equipped with reporter genes, that expressed FiCAR 1 or -2 (Jahan et al., manuscript submitted). Both abundant CD4^+^ (Liadi et al., 2015; Melenhorst et al., 2022) and early T cell memory populations (Louis et al., 2011; Biasco et al., 2021) have been linked to T cell persistence. Moreover, using early memory T cells (for classification: see Table 1.) in *in vivo* tumor models has resulted in improved performance (Sommermeyer et al., 2016). Whether the higher percentage of CD4^+^ cells seen in FiCAR 1 and -2 T cells compared to IgG1-CAR and FiCAR 7 T leads to better persistence *in vivo*, remains to be tested in further studies.

In functional assays involving co-cultures of CAR T cells with CD19^+^ target cells we noticed a small but non-significant trend of higher IL-2 production by FiCAR 1 and -2 T cells compared to those expressing IgG1-CAR and FiCAR 7. Higher IL-2 production may be related to the higher CD4^+^ helper T cell content of the FiCAR 1 and -2 T cells products, as these cells are known to produce most of the cytokines (Malek, 2008). Although the FiCAR 7 and IgG1-CAR T cells contained a higher proportion of cytotoxic CD8^+^ T cells, FiCAR 1 and -2 T cells equipped with the SIRPα spacer displayed equal cytotoxic efficacy. In this setting, IL-2 produced by CD4^+^ T cells might enhance the activation and cytolytic activity of CD8^+^ CAR T cells (Janssen et al., 2003; Liao et al., 2013), explaining the equal cytotoxicity (Figure 3C).

Traditional IgG1-based CARs containing a FcR-binding region interact with different off-target cells including myeloid cells and NK cells, impeding anti-tumor activity of CAR T cells *in vivo* and causing AICD and CAR T cell sequestration in the lungs (Hombach et al., 2010; Almåsbak et al., 2015; Hudecek et al., 2015). We found that in contrast to FiCAR T cells, the IgG1-CAR T cells were activated, were induced to produce IL-2 and IFN-γ, showed a higher proliferation, and displayed off-target cytotoxicity during co-culture with THP-1 monocytes. We hypothesize that the activation of IgG1-CAR T cells observed in the short term co-culture in our experiments may manifest itself as the AICD reported by others in longer term animal studies (Hudecek et al., 2015). Thus, to improve CAR T cell persistence and long-term anti-tumor efficacy, FcR-binding sites must be mutated or removed. Here we have shown that by replacing the (IgG1-) CH2-CH3 region with SIRPα Ig-like C1 domains, we created functional spacers for CARs that do not induce activation, elevated cytokine production or FcR-directed cytotoxicity in T cells nor cytokine production in FcR-expressing THP-1 monocytes (Figure 4). The absence of activation-inducing signaling suggests that there was no adverse off-target binding. Although the functional efficacy was practically identical in all the CARs, there were minor non-significant differences in their memory phenotypes. The causes for the differences in population subtypes remains currently hypothetical but as the culturing conditions remained identical, our results suggest that they may have been caused by CAR-related antigen-independent low level signaling, or interactions of CARs with other signaling moieties.

In short term *in vitro* killing assays, FiCAR 2 T cells displayed equal cytotoxic potential to those equipped with FiCAR 1, FiCAR 7 or IgG1-CAR. However, we observed a small but non-significant increase in the expression of PD-1 and CD57 in the FiCAR 2 T cells. This seems to translate to altered functional properties, as revealed in more thorough *in vitro* and *in vivo* studies (Koski J. et al., manuscript in preparation).

We hypothesize that the FiCAR spacers we have created can be equipped with alternative scFv domains to target other antigens, and that these SIRPα modules can be used to optimize the length of the CAR thus providing the optimal membrane proximity for more complex or secluded target antigens, (Jahan F. et al. submitted; Elmadani M. et al. unpublished). Considering these results, the SIRPα-based spacers represent a promising CAR structure to be used in future *in vivo* models and in clinical applications.

## 4 Materials and methods

### 4.1 Design of the chimeric antigen receptors

The sequences of the FMC63 antibody clone variable regions (Genbank: immunoglobulin light chain, variable region; CAA74660.1 and immunoglobulin heavy chain, variable region; CAA74659.1) were modified to design the CD19 targeting scFv by joining the variable light chain and variable heavy chain with four conventional GGGGS-linkers. The hinge region from IgG1-CH1 -domain was used to join the scFv to the spacer. To design the spacer between the hinge and cell membrane, the length of IgG1-CH2CH3 constant domains [the Protein Data Bank: 1HZH (Saphire et al., 2001)] was measured using software Bodil (version 5203; Mark Johnson, Åbo Akademi University, Finland) and SIRPα Ig-like C1-type 1 and C1-type 2 domains [the Protein Data Bank: 2WNG (Hatherley et al., 2009)] were selected as suitable candidates. The SIRPα primary structure was obtained from the Uniprot database (P78324-1) and considering the optimal expression and codon selection bias, reverse translated using *Homo sapiens* codons by means of estimated probabilities based on frequency distribution (Quax et al., 2015) (CD19-SIRPαCD28ζ, later called FiCARs 1 and -2). The extracellular (in FiCAR 2), transmembrane (TM) and intracellular (IC) sequences were from the T cell-specific surface glycoprotein CD28 (Uniprot P10747-1) and from the intracellular T lymphocyte activation domain of the T cell receptor (TCR, CD3ζ-chain; Uniprot P20963-3).

As a positive control we used an IgG1-based 2nd generation CAR (FMC63 scFv, IgG1-CH2-CH3 spacer, CD28 TM- & IC-domain and CD3ζ-signaling domain; IgG1-CAR; a generous gift from Dr. Gianpietro Dotti, University of North Carolina, United States of America) and as a FcR-binding site free control we used a CD28-based CAR (CD19^−^CD28ζ,; FMC63 scFv, CD28 spacer, TM- & IC-domain, followed by the CD3ζ-signaling domain; called FiCAR 7). T cells transduced with an empty lentiviral vector (mock) were used as negative controls in all production batches.

All sequences were analyzed and assembled *in silico* using the SnapGene® -software (from GSL Biotech; available at snapgene.com). The transgene synthesis and cloning (FiCAR 1, -2 and -7) into the lentiviral vector transfer construct (Schenkwein et al., 2010) was outsourced to GeneArt (Thermo Fisher Scientific GENEART GmbH, Regensburg, Germany). Third generation lentiviral vectors carrying the FiCARs and IgG1-CAR genes and the mock vector were produced in the National Virus Vector Laboratory at the A.I. Virtanen Institute for Molecular Sciences (University of Eastern Finland, Finland).

### 4.2 T cell expansion

CAR T cells were manufactured from three healthy donors as previously described (Kaartinen et al., 2017). Briefly, buffy coats from voluntary donors, not required for treatment of patients, were obtained from the Finnish Red Cross Blood Service under an institutional permit (FRCBS 178/39/2019). Peripheral blood mononuclear cells (PBMC) were separated from 1 day old buffy coats using Ficoll-Paque Premium (GE Healthcare, Chicago, United States) density gradient separation, and T cells were isolated and activated with CD3/CD28 microbeads (Dynabeads Human T-Expander CD3/CD28, Thermo Fisher Scientific, Carlsbad, United States) at a 3:1 microbead to T cell ratio. In T cell cultures, X-VIVO 15 (Lonza, Basel, Switzerland) medium supplemented with 5% human AB-serum (Seralab, Oviedo, Spain) and 100 U/ml of IL-2 (Proleukin, Novartis, Basel, Switzerland) was used. T cell density was adjusted to 1x10^6^ cells/ml on days 0-2 and on day 2 the T cells were transduced using a 3^rd^ generation lentiviral vector containing FiCARs 1, -2, -7, or the IgG1-based CAR or with mock vector. On day 3, after washing off the vector, the T cell density was adjusted to 0,5x10^6^ cells/ml by adding fresh culture medium. CAR T cells were cultured until day 10 and then cryopreserved. For assessing CAR T cell functionality, day 10 CAR T cells were thawed, adjusted to a cell density of 0,5x10^6^ cells/ml and rested in complete media until day 13 before experiments. For memory phenotyping, CAR T cells were cultured until day 13 and analyzed without cryopreservation. The workflow of the experiments is presented in Supplementary Figure S1A.

### 4.3 Cell lines

NALM-6 (CD19^+^ B lineage, ALL) cells were a generous gift from Dr. Olli Lohi (University of Tampere, Finland) and the THP-1 (FcR^+^ monocytes, acute monocytic leukemia) and RS4.11 cell lines were purchased from ATCC (THP-1; TIB-202 and RS4.11; CRL-1873). RS4.11 was transduced using MOI 10 with luc2-eGFP vector (Vectorbuilder, Chicago United States). The NALM-6-luc and Kasumi-2-eGFP-luc cell lines were a generous gift from Dr. Satu Mustjoki and the generation of the luc^+^ cells has been described previously (Dufva et al., 2020). Briefly, Kasumi-2 and NALM-6 cells were transduced with lentiviral vector carrying luciferase and selection marker eGFP (Kasumi-2) or luciferase and neomycin resistance genes (NALM-6) in the presence of polybrene under centrifugation (800 g for 2 h). The resistant NALM-6 cells were selected using G418 disulfate (Sigma-Aldrich, Saint Louis, United States). All the cell lines were sorted with a Sony SH800 cell sorter (Sony Biotechnology, San Jose, United States) and high luciferase-expressing clones were chosen. All the cell lines were cultured in RPMI-1640 medium (Thermo Fisher Scientific, Waltham, United States) supplemented with 10% fetal bovine serum (Thermo Fisher Scientific), 100 IU/ml penicillin and 100 μg/ml streptomycin (Thermo Fisher Scientific).

### 4.4 Flow cytometry

The cells were fixed with 1% paraformaldehyde (10 min, +4°C) prior to staining with antibodies. For analysis of the 48 h co-culture experiments of CAR T cells with THP-1 monocytes, the cells were not fixed. As a control fluorescence minus one (FMO) and/or appropriate isotype controls were used. Samples were run on a BD FACSAria IIu cytometer (BD Biosciences, Franklin Lakes, United States) or with Navios (Beckmann Coulter, Brea, United States) and the results were analyzed using FlowJo (version 10.5.3, BD Biosciences) software.

### 4.5 Chimeric antigen receptor T cell functionality

#### 4.5.1 Memory phenotyping of T cells

T cell subtypes and residual NK- and NKT cells (Table 1) were stained using the following anti-human antibodies from BD Biosciences: CD3 (clone UCHT1)-Fluorescein isothiocyanate (FITC), CD4 (clone SK3)- BD Horizon™ Brilliant Violet™ 510 (BV510), CD8 (RPA-T8)- BD Horizon™ Brilliant Violet™ 421 (BV421), CD56 (clone B159)-Allophycocyanin (APC). Memory T cell phenotypes were identified using CD27 (clone M-T271)-Peridinin-chlorophyll protein (PerCP) conjugated with Cyanine 5.5 (Cy 5.5), CD45RA (clone HI100)-APC, CD45RO (clone UCHL1)- Phycoerythrin (PE) conjugated with Cyanine 7 (Cy7) and CD95 (clone DX2)-PE.

The T cell memory phenotypes were defined using expression markers shown in Table 1 for CD4 and CD8 subpopulations. To specify T cell maturation into a terminal effector-phenotype and exhaustion, antibodies for CD57 (clone NK-1)-BD Horizon™ Brilliant Violet™ 421 (BV421) and CD279 (clone MIH4)-AF647 were used. The expression of T cell terminal effector describing marker CD57 and programmed cell death protein-1 (PD-1/CD279) was assessed in the CD95^+^ CD27 ± CD45RO ± populations. CAR-expression was measured using a F (ab′)2 fragment goat-antihuman immunoglobulin (Ig)G (H + L) conjugated with Alexa Fluor® 647 (Jackson Immunoresearch, Inc. West Grove, United States).

#### 4.5.2 Cytotoxicity assay

To assess the cytotoxic efficacy of CARs, the T cells were co-cultured with Luc^+^ NALM-6, eGFP^+^Luc^+^ Kasumi-2 or Luc2^+^eGFP^+^ RS4.11 cells at various effector: target–ratios (E:T) for 18 h. At the end of the co-culture, luciferin (ONE-Glo Luciferase reagent, Promega, Madison, United States) was added and the presence of live target cells was quantified according to the manufacturer’s instructions with a CLARIOstar Plus Multi-Mode Microplate Reader (BMG Labtech, Ortenberg, Germany). Results were analyzed using CLARIOstar software MARS v5.20 R5.

#### 4.5.3 Degranulation assay

To measure target cell-induced degranulation of T cells, cells were co-cultured with NALM-6 target cells at a 1:1 E:T ratio for 4 h in the presence of a lysosomal-associated membrane protein 1 (CD107a) antibody (PE conjugated, clone H4A3, BD Biosciences) and GolgiStop™ Protein Transport Inhibitor (BD Biosciences). Degranulation was assessed as the proportion of T cells expressing cell surface CD107a^+^ of total T cells in co-cultures, measured by flow cytometry.

#### 4.5.4 Analyses demonstrating chimeric antigen receptor T cell interactions with monocytes

To analyze the effects of CAR T cell binding to monocytes, T cells were co-cultured with the THP-1 monocytic cell line at a 1:1 ratio for 18 h for activation measurements or 48 h for cytotoxicity at +37°C. After 18 h co-culture, the expressions of T cell surface activation markers were analyzed by flow cytometry using CD25-AF647 (clone BC96, BioLegend) and CD69-PE (clone FN50, BD Biosciences) antibodies. To identify cell types, at both timepoints the cells were stained with CD3-FITC and CD32-BV421 (clone FLI8.26, BD Biosciences) or CD64 (clone 10.1, BD Biosciences), and the expression was measured using flow cytometry.

#### 4.5.5 Cytokine assays

To quantify activation induced cytokines from cytotoxicity assays (IFNγ and IL-2) and from analyses demonstrating CAR T cell interactions with monocytes (IFNγ, IL-2 and IL-1β), cell culture media (E:T ratio; 1:1) were analyzed by Cytometric Bead Array (CBA Human Soluble Protein Master Buffer Kit together with IL-2, IFNγ and IL-1β CBA Flex Sets, BD Biosciences) according to the manufacturer’s instructions. The analysis was accomplished using BD FACSAria IIu cytometer (BD Biosciences). Results were analyzed using FCAP Array Software v 3.0 (BD Biosciences).

#### 4.5.6 *In vivo* pilot

A pilot experiment to study the functionality of the FiCAR 1 T cells *in vivo* was carried out using female NSG-mice (The Jackson Laboratory, United States). 0.5 x 10^6^ Luc^+^ NALM-6 cells were engrafted *via* the tail vein into mice on day 0. FiCAR 1 T cells were thawed and subcultured for 6 days (i.e., until day 17 of the whole expansion protocol) followed by injection *via* the tail vein in different doses (0.5, 1.0 and 5.0 million T cells per mouse; two mice per group) 7 days after the target cell injection. Before bioluminescence imaging (BLI), mice were anesthetized using isoflurane (1000 mg/g Attane vet; Piramal Critical Care B.V, Netherlands) and injected intraperitoneally with D-luciferin (25 g/ml; D-luciferin substrate salt, Synchem Germany). After D-luciferin injection, BLI was performed with Lago II (Spectral Instruments imaging, United States) and the total emission values were collected using software Aura version 3.1.0 (Spectral Instruments imaging, Arizona, United States). All animals were handled according to approved institutional Animal Care and Use committee protocols of the University of Helsinki. The protocol was approved by the Regional State Administrative Agency for Southern Finland (ESAVI/10548/2019).

### 4.6 Statistics and figures

For statistical analyses SPSS (version 27.0.1.0) software was used. The possible correlations between donors and CAR T cell related variables were analyzed using Spearman’s rho two-tailed test and the variances between single groups were studied with Independent *t* Test, Mann-Whitney *U* Test or one-way ANOVA and multiple comparison with Bonferroni correction. Differences between fold cumulative expansion were analyzed by means of non-linear regression model and one-way ANOVA. Differences were considered statistically significant when *p* < 0.05. All the figures were graphed using GraphPad Prism (version 8.0.2) and illustrations created with BioRender.com.

## Data Availability

The original contributions presented in the study are included in the article/Supplementary Material, further inquiries can be directed to the corresponding author.
