# Supplementary material for: Novel modular chimeric antigen receptor spacer for T cells derived from signal regulatory protein alpha Ig-like domains
Source: Front Mol Med. 2022 Dec 13;2:1049580. doi: 10.3389/fmmed.2022.1049580 (PMC11285650; doi:10.3389/fmmed.2022.1049580)
Supplement: Supplementary file 1 [file Presentation1.pdf]

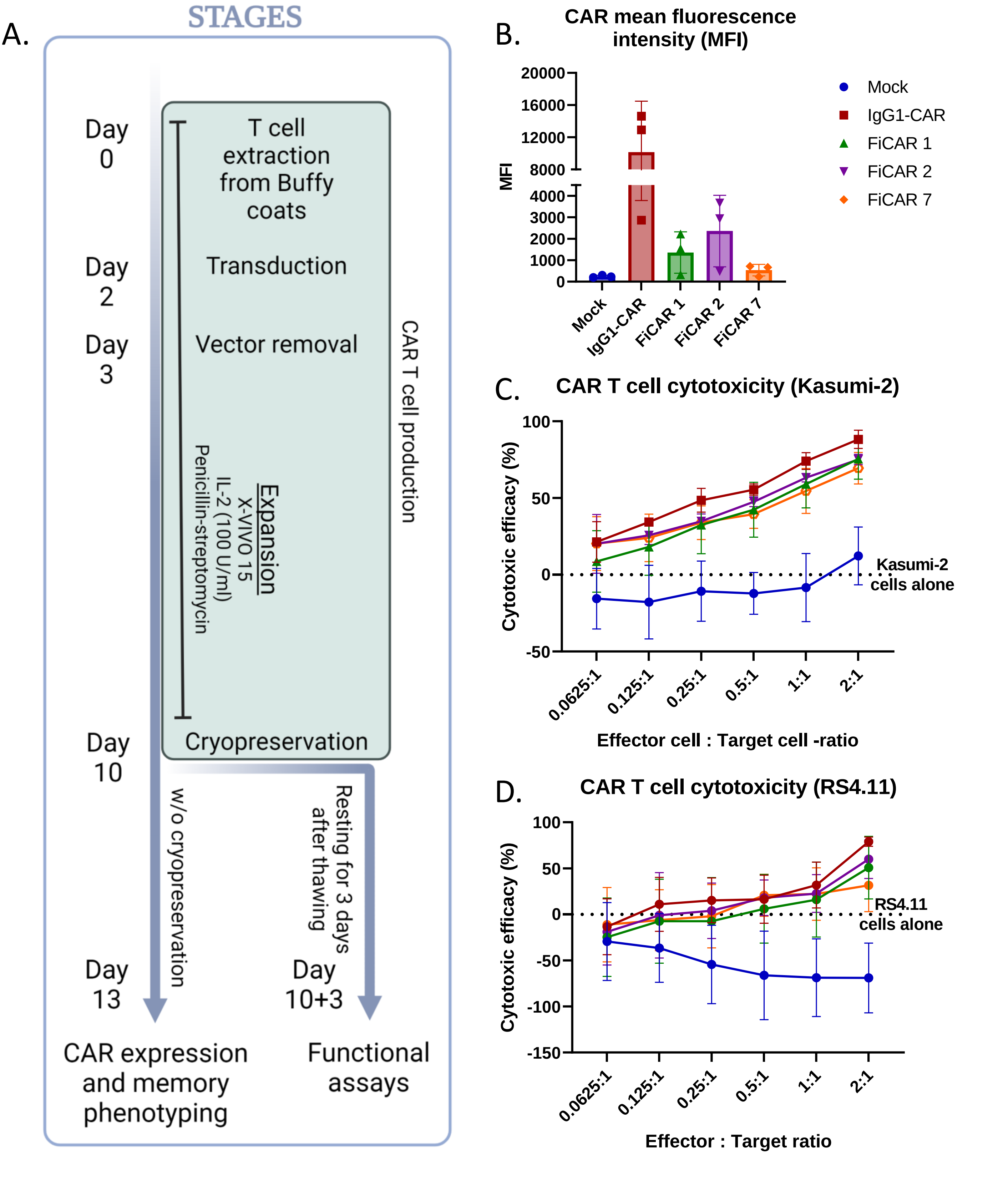

Supplementary Figure 1. **Experimental workflow and CAR T cell characterization.** A. CAR T cell expansion and the related steps are illustrated in a timeline. B. Mean fluorescence intensity values of different CAR T cells measured by flow cytometry on day 13 of culture. Results obtained from CAR T cells from individual donors (n = 3) are shown with individual symbols, and the mean values and standard deviations with bars and lines, respectively. C. and D. CAR T cell cytotoxicity against $CD19^+$ ALL cell lines $eGFP^+Luc^+$ Kasumi-2 and $Luc2^+eGFP^+$ RS4.11 at various E:T ratios. Data points and horizontal lines show the mean values +/- SD (n=3).

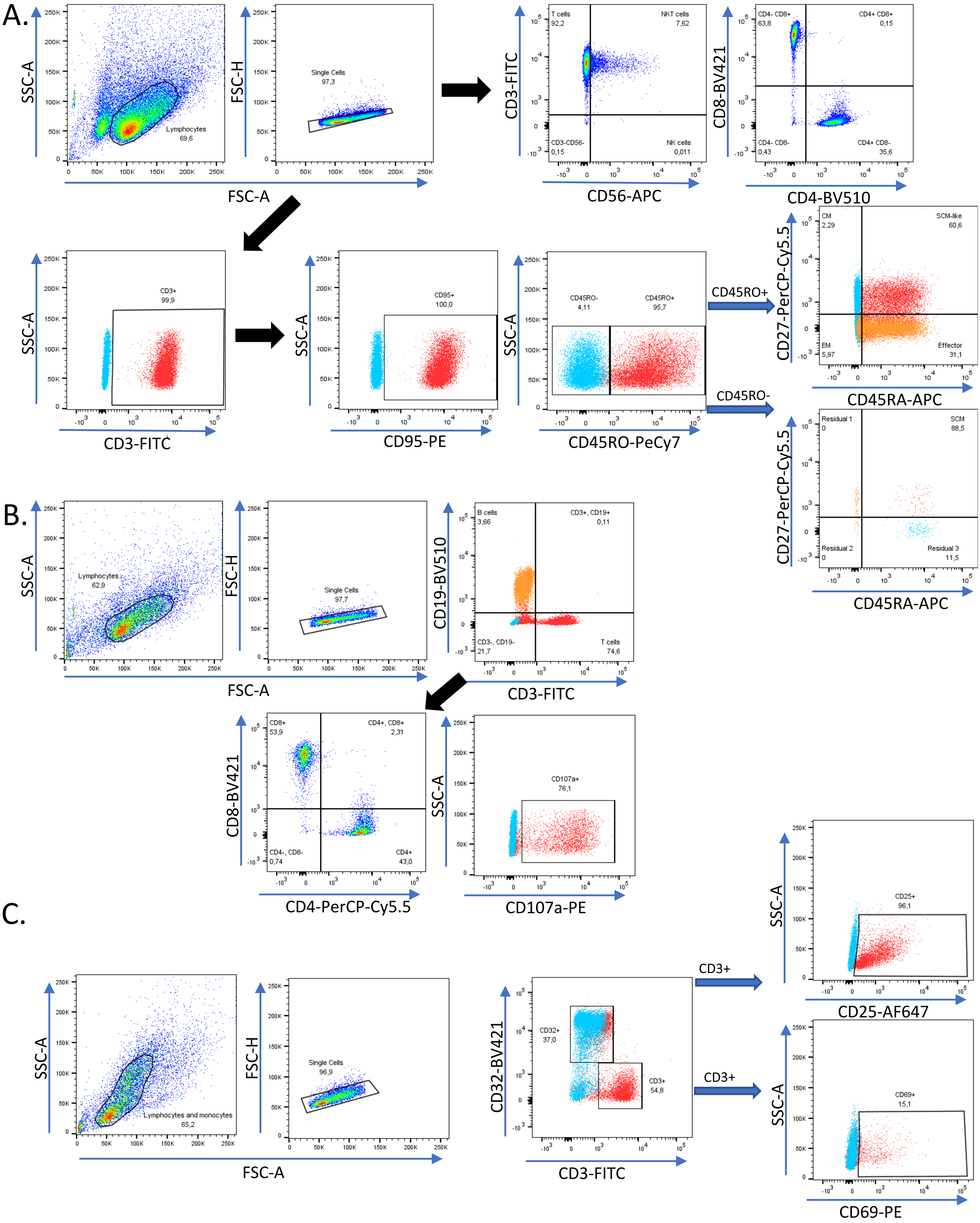

Supplementary Figure 2. **Exemplary gating strategies identifying different cell types or analyzed cell surface markers in flow cytometry.** Exemplary gating strategy in flow cytometry with isotype or FMO controls. Colors represent isotype or FMO control in cyan and sample in red to identify **A.** T, NK and NKT cells **;** T cell memory phenotypes **B.** degranulation of T cells or **C.** activation markers CD25 and CD69 in T cells in T cell + THP-1 monocyte co-cultures.
